# Pharmacological and Therapeutic Potential of Garlic (*Allium sativum*): Integrating Phytochemistry, Molecular Mechanisms, and Clinical and Preclinical Evidence

**DOI:** 10.1002/open.70321

**Published:** 2026-10-27

**Authors:** Gudisa Bereda

**Affiliations:** ^1^ Department of Pharmacy Marie Stopes International Ethiopia (MSIE) Ambo Ethiopia

**Keywords:** *Allium sativum*, clinical studies, garlic, immunomodulation, preclinical studies, therapeutic effects

## Abstract

Garlic (*Allium sativum*) is a medicinal plant with diverse pharmacological properties and therapeutic potential in metabolic, cardiovascular, hepatic, immune, inflammatory, infectious, and neoplastic disorders. However, comprehensive integration of clinical and preclinical evidence remains limited. This review synthesized randomized controlled trials, crossover studies, therapeutic clinical investigations, and experimental animal studies evaluating garlic supplementation. Allicin was identified as the principal intermediate precursor generating numerous bioactive organosulfur compounds. Clinical evidence demonstrated reductions in systolic blood pressure of 5.2%–10.5% and diastolic blood pressure of 4.0%, decreases in LDL cholesterol of 11.8%–14.2% and total cholesterol of 7.6%–14.2%, and increases in HDL cholesterol of 11.5%. Fasting glucose decreased by 7.01 mg/dL, and HbA1c by 0.66%. Inflammatory markers, including TNF‐α and C‐reactive protein, declined by up to 24.7%, whereas antioxidant capacity increased. Preclinical studies demonstrated reduced tumor growth, improved renal function, enhanced epithelial regeneration, and increased survival in experimental infection models. Garlic demonstrates consistent therapeutic benefits across multiple conditions. Nevertheless, additional high‐quality, large‐scale clinical trials are required to establish optimal dosing, long‐term safety, and clinical efficacy.

## Graphical abstract

Garlic bioactive compounds, including organosulfur compounds (allicin, DADS, DATS, and SAC), phenolic compounds, and quercetin, regulate the Nrf2/HO‐1, NF‐κB, PI3K/Akt, and AMPK/eNOS signaling pathways. These actions enhance antioxidant defenses, suppress inflammation, improve metabolic and cardiovascular function, and contribute to reduced blood pressure, improved lipid profiles, lower pain, enhanced oxidative status, and better exercise recovery.

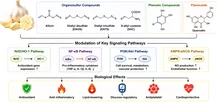

## 1 Introduction

Garlic (*Allium sativum* L.) is one of the most widely cultivated medicinal crops worldwide [1]. It is highly valued for its nutritional and therapeutic properties. It has been used for more than 5000 years in ancient civilizations such as Egypt, Greece, China, and India. In these societies, it was used for infections, gastrointestinal disorders, and cardiovascular diseases [2]. Traditional medical systems, including Ayurveda and Traditional Chinese Medicine, describe garlic as a substance that enhances immune function [3]. It also reduces inflammation and improves metabolic balance. The regular consumption of garlic reduces total cholesterol by approximately 10% [4]. It also improves the HDL‐to‐LDL ratio by increasing HDL cholesterol and reducing LDL cholesterol. However, these effects vary depending on diet and population differences.

From a nutritional perspective, fresh garlic contains approximately 63% water [5]. It also contains 28% carbohydrates, in the form of fructans. In addition, it contains 2.3% organosulfur compounds, 2% protein (including alliinase), 1.2% free amino acids such as arginine, and 1.5% fiber. Similar compositional profiles have been reported in other analyses [5]. These studies document approximately 65% water and 28% carbohydrates. They also report 2.3% organosulfur compounds, 2% protein, 1.2% free amino acids, and 1.5% fiber in garlic. Bulbs of *Allium sativum* contain a wide range of bioactive compounds. Sulfur‐containing molecules are the most dominant group [6]. These include thiosulfinates such as allicin. They also include sulfides such as diallyl disulfide (DADS) and diallyl trisulfide (DATS). Other compounds include vinyldithiins (2‐vinyl‐(4H)‐1,3‐dithiin and 3‐vinyl‐(4H)‐1,2‐dithiin), ajoenes (E‐ajoene and Z‐ajoene), and S‐allyl cysteine (SAC) [6].

From a phytochemical and mechanistic perspective, allicin is the primary bioactive compound [7]. It is formed when alliin is enzymatically converted after garlic is crushed. However, allicin is chemically unstable. It rapidly transforms into secondary metabolites. These include DADS, DATS, ajoene, and SAC [8]. These organosulfur constituents represent the most pharmacologically active fraction of garlic. They are responsible for many of its biological effects. Fresh garlic cloves contain the highest allicin concentration, approximately 12 mg g^−1^ dry weight (DW) [9]. However, processing significantly reduces this content. Freeze‐drying results in an approximate 15% reduction. Oven‐drying at 55 °C leads to about a 40% reduction. Drying at 70 °C results in approximately a 70% reduction [9].

In addition, thermal processing affects other phytochemicals. Fresh garlic extracts demonstrate total phenolic contents of 303.07 ± 6.58 mg GAE/100 g (aqueous) and 638.96 ± 15.30 mg GAE/100 g (ethanolic) [10]. Flavonoid contents are 109.78 ± 6.78 mg QE/100 g (aqueous) and 258.47 ± 12.37 mg QE/‍100 g (ethanolic). FTIR analysis confirms the presence of carboxylic acids, hydroxyls, esters, and benzene functional groups [10]. These indicate the presence of phenols and flavonoids. Cooking temperature significantly alters these compounds. However, cooking time has no significant effect on phytochemicals or antioxidant capacity. Pharmacologically, garlic exhibits multiple cardiometabolic benefits. Supplementation improves lipid profile, glycemic control, and blood pressure [11]. It also reduces inflammation and oxidative stress. However, it has no significant effect on apolipoproteins, certain cytokines, body weight, or adiponectin levels. These cardioprotective effects are mainly attributed to multiple mechanisms. These include inhibition of cholesterol biosynthesis by modulating HMG‐CoA reductase [11]. They also include enhanced nitric oxide production and reduced platelet aggregation.

In terms of antioxidant activity, garlic supplementation significantly increases total antioxidant capacity (TAC) and superoxide dismutase (SOD) by 2.77 and 13.76 units, respectively [12]. It also reduces malondialdehyde (MDA) levels by 1.94. These findings demonstrate its ability to enhance endogenous antioxidant defense systems. The anti‐inflammatory effects of garlic are primarily mediated through inhibition of key intracellular signaling pathways, particularly NF‐κB [13]. This suppression leads to reduced production of proinflammatory cytokines such as tumor necrosis factor‐alpha (TNF‐α) and interleukin‐6 (IL‐6). These mechanisms collectively contribute to its systemic anti‐inflammatory activity. Additionally, garlic exhibits broad‐spectrum antimicrobial activity. In vitro studies demonstrate that low concentrations of allicin can inhibit both Gram‐positive and Gram‐negative bacteria [2]. This supports its traditional use in infection control.

Similar to other medicinal plants [14, 15, 16], comprehensive evaluation of *Allium sativum* L. requires integration of traditional knowledge, phytochemistry, pharmacological evidence, and safety assessment to clarify its therapeutic potential. Despite these promising findings, the clinical translation of garlic’s therapeutic effects remains inconsistent. Clinical studies in healthy subjects have shown that standardized garlic supplementation (900 mg/day) can significantly reduce total cholesterol (TC) and low‐density lipoprotein cholesterol (LDL‐C) levels [17]. However, the clinical utility of garlic is limited by the poor bioavailability, rapid metabolism, and chemical instability of key bioactive compounds such as allicin. These limitations also complicate dose standardization and reduce comparability across clinical studies. This narrative review provides a comprehensive synthesis of garlic phytochemistry, experimental evidence, and clinical outcomes, with particular emphasis on mechanistic pathways and translational relevance. It integrates preclinical findings with human clinical studies and considers dose–response relationships. In doing so, it bridges experimental evidence with observed clinical effects. This framework supports a structured evaluation of garlic’s therapeutic potential in cardiometabolic health, immune modulation, renal function, and oxidative stress‐related conditions.

## 2 Materials and Methods

### 2.1 Review Design and Framework

This narrative review was conducted in accordance with the Assessment of Narrative Review Articles (SANRA) guidelines [18] to critically synthesize clinical, preclinical, and mechanistic evidence regarding the therapeutic effects and pharmacological activities of *Allium sativum* L. (Figure S1).

### 2.2 Literature Search Strategy

A literature search was conducted in PubMed, Scopus, Web of Science, and Google Scholar for publications available from March 2000 to March 2026. The search strategy included keywords and Medical Subject Headings (MeSH) terms related to garlic and its therapeutic effects, including “Garlic,” “*Allium sativum*,” “therapeutic effects,” “pharmacological activity,” “cardiometabolic effects,” “renal protection,” “immunomodulation,” “antioxidant,” “anti‐inflammatory,” “clinical studies,” and “preclinical studies.” Boolean operators (AND/OR) were applied to refine the search strategy.

### 2.3 Eligibility Criteria

Studies were selected based on predefined inclusion and exclusion criteria. Eligible studies included original research articles, randomized controlled trials (RCTs), clinical trials, observational studies, systematic reviews, meta‐analyses, animal experiments, and in vitro studies evaluating the therapeutic, biological, or pharmacological effects of garlic or garlic‐derived preparations. Studies were excluded if they were unrelated to therapeutic outcomes, lacked primary data, or were conference abstracts, editorials, letters without original findings, duplicate publications, or non‐English articles.

### 2.4 Study Selection and Data Extraction

Articles were screened initially by title and abstract, followed by full‐text evaluation to confirm eligibility. Data were extracted on study design, population or experimental model, garlic preparation, dose, duration, outcomes, safety findings, and mechanisms of action. For preclinical studies, extracted information included species, experimental model, route of administration, dosage, and biological endpoints. For clinical studies, participant characteristics, intervention type, dose, duration, clinical outcomes, and safety findings were recorded.

### 2.5 Evidence Synthesis and Critical Interpretation

As a narrative review, quantitative meta‐analysis was not performed. Findings were synthesized qualitatively and organized according to major therapeutic domains. Evidence was interpreted according to methodological strength, distinguishing systematic reviews and meta‐analyses, randomized clinical trials, observational studies, animal studies, and in vitro investigations. Conflicting findings were critically assessed by considering differences in garlic formulation, standardization of bioactive constituents, dosage, intervention duration, disease characteristics, sample size, and methodological quality. The chemical structures were searched in PubChem (https://pubchem.ncbi.nlm.nih.gov/substance/481107917).

## 3 Results

### 3.1 Phytochemical Composition and Chemical Characteristics

*Allium sativum* has a complex phytochemical profile comprising sulfur‐containing compounds, phenolics, flavonoids, saponins, carbohydrates, proteins, peptides, volatile constituents, lipids, vitamins, and trace elements [19, 20]. Sulfur‐containing constituents represent the characteristic chemical fraction of garlic and contribute substantially to its distinctive odor and reported biological activities [21] (Table 1; Figure 1).

**TABLE 1:** Phytochemical composition, chemical characteristics, formation pathways, and major biological activities of *Allium sativum* constituents.

Phytochemical category	Subclass	Key constituents	Chemical nature/structural features	Origin/formation pathway	Major biological activities	References
Organosulfur compounds	Sulfoxide precursors	Alliin, MCSO, PCSO	Cysteine‐sulfoxide derivatives; nonvolatile sulfur‐containing precursors	Stored in intact bulbs and compartmentalized from alliinase; tissue disruption enables enzymatic conversion.	Precursors of the bioactive sulfur cascade	[22, 23]
Organosulfur compounds	Thiosulfinates	Allicin (diallyl thiosulfinate)	Highly reactive thiosulfinate containing S═O and allyl groups; chemically unstable	Formed when tissue disruption brings alliin into contact with alliinase, generating allylsulfenic acid that condenses to allicin	Antimicrobial, antioxidant, anti‐inflammatory, redox‐related effects; precursor of downstream sulfur compounds	[20–23]
Organosulfur compounds	Lipophilic sulfur derivatives	DAS, DADS, DATS	Allyl sulfide, disulfide, and trisulfide structures containing one, two, and three sulfur atoms, respectively; lipophilic and volatile	Generated mainly through decomposition and thermal/chemical transformation of allicin	Anticancer, cardioprotective, antimicrobial, lipid‐regulatory effects	[20, 23]
Organosulfur compounds	Rearrangement products	E/Z‐ajoene, vinyldithiins	Ajoene contains sulfur‐linked unsaturated groups; vinyldithiins are cyclic sulfur‐containing compounds; generally lipophilic.	Formed through allicin rearrangement during processing, particularly in oil‐ or solvent‐associated conditions	Antithrombotic, antimicrobial, anticancer effects	[23, 24]
Organosulfur compounds	Water‐soluble aged‐garlic derivatives	SAC, SAMC, N‐acetylated derivatives	Stable cysteine‐derived sulfur compounds with an allyl‐sulfur moiety; hydrophilic	Accumulate during aging or fermentation of garlic preparations	Antioxidant, neuroprotective, hepatoprotective, anti‐inflammatory, antiproliferative effects	[23, 25, 26, 27, 28]
Phenolic compounds	Phenolic acids	Ferulic acid, p‐coumaric acid, gallic acid derivatives	Aromatic phenolic structures containing hydroxyl groups; generally polar to moderately polar	Plant phenylpropanoid‐related metabolism	Antioxidant, anti‐inflammatory, anticancer effects	[29, 30]
Phenolic compounds	Flavonoids	Quercetin, kaempferol	Polyphenolic flavonoid structures with multiple hydroxyl groups	Plant secondary metabolism	Free‐radical scavenging, antioxidant, anti‐inflammatory effects	[29, 30]
Saponins	Steroidal saponins	Steroidal glycosides	Steroid backbone conjugated with glycosidic sugar chains; amphipathic	Secondary plant metabolism and steroid‐backbone glycosylation	Antifungal, membrane‐modulating, immunomodulatory, and lipid‐regulatory effects	[28, 31]
Proteins and peptides	Enzymatic and bioactive peptides	Alliinase, antimicrobial peptides	Proteins and peptide molecules with diverse structural characteristics	Protein biosynthesis; alliinase is stored separately from its substrate in intact tissue	Enzymatic activation of sulfur precursors; antimicrobial defense	[32]
Carbohydrates	Fructan polysaccharides	Inulin‐type fructans	Water‐soluble fructose polymers, predominantly containing β‐(2→1) linkages.	Storage carbohydrate metabolism in garlic bulbs	Prebiotic effects, gut microbiota modulation, immunomodulatory and metabolic effects	[33]
Volatile compounds	Low‐molecular‐weight sulfur volatiles	DAS, DADS and related volatile sulfur compounds	Small, sulfur‐containing, relatively volatile molecules	Decomposition and transformation of organosulfur constituents	Aroma, antimicrobial effects, and signaling‐related activities	[23]
Lipids	Fatty acid fraction	Minor fatty acids	Fatty acid molecules forming part of the lipid fraction	Plant lipid biosynthesis and metabolism	Membrane integrity and signaling modulation	[34]
Micronutrients	Vitamins and trace elements	Vitamin C, B‐complex vitamins, selenium	Low‐molecular‐weight vitamins and essential trace elements	Plant metabolism and mineral uptake	Nutritional functions, antioxidant support, and redox homeostasis	[22]

**FIGURE 1:**
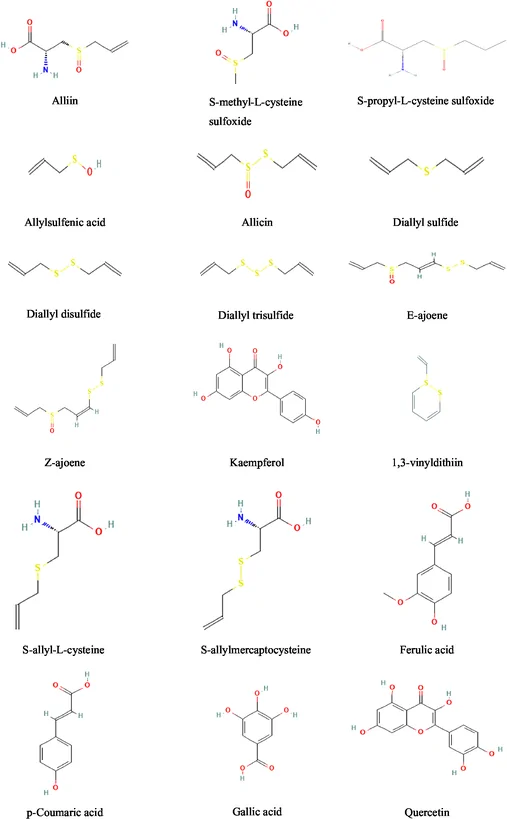
Major phytochemical constituents and chemical transformation pathways of *Allium sativum*.

### 3.2 Organosulfur Precursors and Enzymatic Activation

In intact garlic bulbs, the principal sulfur‐containing precursors are S‐alk(en)yl‐L‐‍cysteine sulfoxides, particularly S‐allyl‐L‐‍cysteine sulfoxide (alliin), together with S‐methyl‐L‐cysteine sulfoxide (MCSO) and S‐propyl‐L‐cysteine sulfoxide (PCSO) [22]. These precursors are compartmentalized from alliinase within undamaged tissues. Alliin is an amino acid‐derived sulfoxide with the molecular formula C_6_H_11_NO_3_S and a molecular weight of 177.22 g/mol [35]. Its amino and carboxyl groups contribute to its polar and acid–base characteristics, whereas its sulfoxide group represents the principal sulfur‐containing structural feature. Reported physicochemical descriptors include an XlogP3‐AA value of approximately −3.5, two hydrogen‐bond donors, five hydrogen‐bond acceptors, and a topological polar surface area of approximately 99.6 Å^2^ [36]. Mechanical disruption of garlic tissue brings alliin into contact with alliinase [37]. The enzyme catalyzes the cleavage of alliin to allylsulfenic acid, pyruvate, and ammonia [23]. The resulting allylsulfenic acid undergoes condensation to form allicin. This precursor–enzyme system accounts for the rapid chemical changes that occur when garlic is cut, crushed, or otherwise disrupted.

### 3.3 Allicin and Allicin‐Derived Organosulfur Compounds

Allicin, or diallyl thiosulfinate, is a principal transient sulfur‐containing constituent of disrupted garlic [38]. It has the molecular formula C_6_H_10_OS_2_ and a molecular weight of approximately 162.3 g/mol [35]. The compound contains a thiosulfinate functional group with allyl substituents and represents a central intermediate in the formation of numerous downstream organosulfur metabolites [39].

The allicin‐derived group includes diallyl sulfide (DAS), diallyl disulfide (DADS), diallyl trisulfide (DATS), E‐ and Z‐ajoene, and 1,2‐ and 1,3‐vinyldithiins [40]. DAS, DADS, and DATS contain allyl groups associated with one, two, and three sulfur atoms, respectively, and constitute important oil‐soluble sulfur compounds [20, 23]. Ajoenes and vinyldithiins represent structurally distinct sulfur‐containing transformation products [23, 24]. Transformations involving MCSO and PCSO further contribute to the diversity of thiosulfinates and polysulfides. More than 50 sulfur‐containing metabolites have been reported under different conditions, highlighting the chemical diversity of garlic sulfur metabolism and processing [41].

### 3.4 Water‐Soluble Sulfur Compounds

Water‐soluble sulfur compounds constitute an important fraction of aged garlic preparations. These include S‐allyl‐L‐cysteine (SAC), S‐allylmercaptocysteine (SAMC), and N‐acetylated derivatives, which accumulate during the aging process [25]. SAC is a cysteine‐derived compound with a comparatively stable hydrophilic structure [42]. Its amino‐acid framework contributes to its aqueous solubility and makes it an important chemical constituent of aged garlic products. SAC is among the principal relatively stable sulfur compounds found in aged garlic and can provide a more sustained source of sulfur‐containing constituents than highly transient compounds present after fresh tissue disruption [23, 26, 27]. SAMC is a related water‐soluble derivative that has been investigated for biological effects, including antiproliferative activity [28].

### 3.5 Nonsulfur Phytochemicals

The nonsulfur fraction of garlic includes phenolics, flavonoids, saponins, fructans, proteins, peptides, lipids, vitamins, and trace elements [43]. Phenolic compounds include ferulic acid, p‐coumaric acid, gallic acid derivatives, and related constituents [44]. Their aromatic rings and hydroxyl groups contribute to reported radical‐scavenging and antioxidant properties [29, ‍30]. Flavonoids such as quercetin and kaempferol provide additional polyphenolic constituents associated with antioxidant and anti‐inflammatory activities [29, 30]. Steroidal saponins comprise glycosylated steroidal aglycones and sugar residues and have been associated with diverse biological effects, including modulation of membrane‐related processes and lipid metabolism [31]. Fructans, characterized predominantly by β‐‍(2→1)‐linked fructose units, serve as major storage carbohydrates and may exert prebiotic and immunomodulatory effects [33]. Proteins and peptides include alliinase and other peptide constituents with reported antimicrobial properties [32]. Fatty acids contribute to the lipid fraction, while volatile constituents include sulfur‐containing compounds such as DAS [23]. Vitamins, including vitamin C and B‐complex vitamins, and trace elements such as selenium contribute to the nutritional and antioxidant‐related profile of garlic [22].

## 4 Physicochemical Properties, Chemical Stability, and Structure–Activity Relationships of *Allium sativum*

The chemical behavior of garlic constituents is influenced by their physicochemical properties, chemical reactivity, stability, and transformations during processing and storage [45]. These characteristics and their relationship to molecular structure and preparation type are important for interpreting differences in the biological properties and reproducibility of garlic‐derived products (Table 2).

**TABLE 2:** Chemical characteristics, physicochemical properties, reactivity, and stability of major bioactive constituents of *Allium sativum*.

Compound	Molecular formula	Molecular weight, g/mol	Structural class and principal functional groups	Key physicochemical descriptors	Acid–base characteristics	Principal chemical reactivity	Stability and chemical transformation	References
Alliin (S‐allyl‐L‐cysteine sulfoxide)	C_6_H_11_NO_3_S	177.22	Cysteine sulfoxide; α‐amino acid containing amino, carboxylic acid, sulfoxide, and allyl groups	XlogP3‐AA − 3.5; HBD 2; HBA 5; TPSA 99.6 Å^2^	Amphoteric amino‐acid derivative; amino and carboxyl groups undergo pH‐dependent protonation/deprotonation	Relatively less chemically reactive than allicin; undergoes enzyme‐catalyzed cleavage by alliinase after tissue disruption.	Relatively stable in intact garlic; alliinase‐mediated conversion produces allylsulfenic acid, which subsequently forms allicin.	[27, 45, 46]
Allicin (diallyl thiosulfinate)	C_6_H_10_OS_2_	162.3	Thiosulfinate containing allyl groups and S–S/S–O sulfur functionality	XlogP3‐AA 1.3; HBD 0; HBA 3; TPSA 61.6 Å^2^	No conventional amino or carboxyl groups; chemistry is dominated by sulfur‐centered reactivity rather than classical acid–base ionization	Highly electrophilic sulfur center; reacts readily with thiol‐containing nucleophiles, including cysteine residues and glutathione, producing sulfur‐transfer/S‐thioallylation products.	Chemically unstable; undergoes decomposition and transformation into ajoenes, vinyldithiins, DADS, DATS, and other sulfur compounds	[20, 40, 45]
E‐Ajoene	C_9_H_14_OS_3_	234.4	Ajoene; sulfoxide and disulfide/polysulfide‐containing structure with allyl groups; E configuration	XlogP3‐AA 1.7; HBD 0; HBA 4; TPSA 86.9 Å^2^	No conventional amino/carboxyl groups; sulfur‐centered chemistry predominates.	Sulfur‐containing structure permits interactions with nucleophilic biological targets.	Secondary transformation product of allicin; abundance depends on processing and storage conditions	[22, 45, 47]
Z‐Ajoene	C_9_H_14_OS_3_	234.4	Geometric isomer of E‐ajoene; sulfoxide and disulfide/polysulfide functionality	Physicochemical behavior broadly related to E‐ajoene; geometric configuration alters molecular arrangement and potentially biological interactions.	No conventional amino/carboxyl groups; sulfur‐centered chemistry predominates.	Sulfur‐containing functional groups can participate in interactions with biological nucleophiles.	Formed during allicin transformation; relative abundance depends on processing conditions.	[22, 45, 47]
Diallyl sulfide	C_6_H_10_S	114.21	Thioether containing two allyl groups	XlogP3‐AA 2.2; HBD 0; HBA 1; TPSA 25.3 Å^2^	No conventional ionizable amino or carboxyl groups	Comparatively low direct sulfur reactivity; thioether sulfur can undergo oxidation and metabolic transformation	More chemically persistent than allicin; occurs in garlic oil and other processed garlic preparations	[22, 46, 48]
Diallyl disulfide	C_6_H_10_S_2_	146.3	Organic disulfide containing two allyl groups and an S–S bond	XlogP3‐AA 2.2; HBD 0; HBA 2; TPSA 50.6 Å^2^	No conventional amino/carboxyl acid–base functionality	Disulfide sulfur can participate in thiol–disulfide exchange and sulfur‐transfer reactions	More persistent than allicin but remains chemically transformable; important constituent of garlic oil	[20, 22, 48]
Diallyl trisulfide	C_6_H_10_S_3_	178.3	Organic trisulfide containing two allyl groups and an S–S–S chain	XlogP3‐AA 2.6; HBD 0; HBA 3; TPSA 75.9 Å^2^	No conventional amino/carboxyl acid–base functionality	Polysulfide sulfur atoms can participate in sulfur‐transfer reactions and interactions with thiol‐containing biomolecules	Occurs among garlic‐derived sulfur compounds and can arise during transformation of other organosulfur constituents; stability depends on chemical matrix and conditions	[20, 22, 48]
S‐Allyl‐L‐cysteine	C_6_H_11_NO_2_S	161.22	Cysteine derivative containing amino, carboxylic acid, thioether, and allyl groups	More polar and water‐compatible than hydrophobic diallyl sulfides	Amphoteric amino‐acid derivative; amino and carboxyl groups exhibit pH‐dependent ionization	Less electrophilically reactive than allicin because sulfur occurs as a thioether rather than a thiosulfinate	Relatively stable compared with allicin; accumulates during garlic aging and is characteristic of aged garlic preparations.	[22, 27, 45]

### 4.1 Physicochemical Properties and Chemical Reactivity

The physicochemical behavior of garlic constituents varies according to molecular polarity, hydrogen‐bonding capacity, sulfur oxidation state, molecular size, and lipophilicity [47]. Alliin and SAC are comparatively polar because of their amino‐acid‐derived structures, whereas allicin, ajoenes, vinyldithiins, DAS, DADS, and DATS have greater compatibility with hydrophobic environments [46]. These differences govern properties such as aqueous distribution, membrane association, cellular permeability, and intermolecular interactions. Alliin and SAC possess amino and carboxyl functionalities and therefore exhibit acid–base behavior associated with amino‐acid structures [27]. Their ionization can influence aqueous distribution and molecular interactions. In contrast, allicin and the diallyl sulfides lack these conventional ionizable groups, and their chemical behavior is primarily determined by sulfur‐centered reactivity and redox chemistry [49].

Allicin contains an electrophilic sulfur center within its thiosulfinate group [40]. It can therefore react with nucleophilic thiol groups, including cysteine residues in proteins and glutathione. These reactions can result in S‐thioallylation and modification of redox‐sensitive proteins and microbial enzymes. Its allyl groups also favor interaction with lipid environments and biological membranes. The high chemical reactivity responsible for these interactions is closely associated with its limited persistence [20]. The number and arrangement of sulfur atoms also influence the reactivity of DAS, DADS, and DATS. Increasing sulfur‐chain length alters sulfur‐transfer behavior, redox characteristics, lipophilicity, volatility, and interactions with thiol‐containing targets and membranes [48]. Differences in sulfur connectivity and molecular geometry likewise affect the chemical behavior of ajoenes and vinyldithiins [47].

### 4.2 Chemical Stability and Processing‐Dependent Transformations

Garlic chemistry is highly dependent on processing conditions [45]. Heating can accelerate chemical decomposition and redirect the distribution of downstream sulfur metabolites [50]. Steam distillation and oil extraction favor recovery of different volatile or lipid‐associated constituents, whereas oil maceration promotes the accumulation of particular transformation products, including vinyldithiins and ajoenes [51]. Drying and powder production can modify enzymatic activity and alter the retention or loss of individual sulfur constituents [27]. Depending on processing conditions, garlic powder may retain substantial amounts of alliin, while volatile compounds can be reduced or redistributed [7].

Aging produces a distinct chemical profile characterized by progressive enrichment of stable water‐soluble sulfur derivatives [22]. Storage can subsequently modify constituent concentrations through changes associated with temperature, moisture, oxygen exposure, solvent environment, and duration [45]. Cysteine and other amino‐acid components within the matrix may also affect the persistence and degradation of reactive sulfur constituents [22]. Thus, processing does not merely preserve or concentrate the constituents originally present in garlic [27]. It can change their relative abundance and generate a chemically different preparation. This variability must be considered when evaluating the composition and stability of garlic‐derived products.

### 4.3 Structure–Activity Relationships and Chemical Distinction Among Garlic Preparations

Structural variation among garlic constituents provides an important basis for differences in biological activity [52]. The relevant structural determinants include sulfur oxidation state, sulfur connectivity, number of sulfur atoms, molecular geometry, functional‐group composition, and overall molecular polarity [53]. These features determine how individual compounds interact with biological molecules and how readily they can reach relevant sites of action. Consequently, structurally related sulfur compounds may exhibit different biological profiles despite originating from the same plant [54]. Chemical composition also determines whether findings obtained with an isolated constituent can be extrapolated to a garlic preparation [55]. Fresh garlic, dried powder, garlic oil, oil‐macerated products, and aged garlic extract represent chemically different matrices rather than interchangeable sources of the same constituents [22]. Their biological effects therefore reflect differences in constituent composition, relative abundance, chemical persistence, dose, absorption, metabolism, and matrix interactions.

For this reason, studies of garlic should report the preparation type and, where possible, its relevant chemical composition rather than relying solely on the botanical identity of the source material [45]. Comparisons of efficacy, safety, pharmacokinetics, and reproducibility should account for these compositional differences [22]. This approach is particularly important when evidence from purified compounds, extracts, and whole‐garlic preparations is considered within the same pharmacological framework.

## 5 Pharmacological Activities

Garlic has been widely studied for its numerous pharmacological benefits [56]. These effects are mainly attributed to its high content of sulfur‐containing compounds, such as allicin, diallyl sulfides, and S‐allyl cysteine. Garlic also contains other bioactive molecules, including flavonoids and phenolic compounds. Together, these compounds contribute to a variety of therapeutic actions. Garlic provides cardiovascular protection, antimicrobial activity, anticancer effects, neuroprotection, metabolic regulation, and immunomodulatory functions [30] (Tables 3 and 4).

**TABLE 3:** Clinical human studies of garlic.

Study design	Population/sample size	Dose	Duration	Main outcomes	Findings	Limitations	References
Single‐blind crossover	11 healthy males	Garlic 1000 mg/day	4 weeks	Oxidative stress & inflammation during cycling	↑ TAC, ↓ MDA, ↓ TNF‐α, ↓ LDH (*p* < 0.05); no effect on glucose, NEFA, IL‐6	Small sample size; healthy population; short duration	[57]
Randomized double‐blind placebo‐controlled	80 post‐menopausal overweight/obese women	Garlic 1000 mg/day	12 weeks	Knee osteoarthritis, cytokines	↓ Resistin (6.41 → 5.56 ng/mL, *p* = 0.008), pain 6.8 → 5.3 (*p* = 0.002); TNF‐α NS	Limited sample size; short intervention period	[58]
Randomized triple‐blind placebo‐controlled	60 women with endometriosis	Garlic 400 mg/day	3 months	Pain severity	Pain 6.51 → 1.83 (*p* < 0.05); Control 6.41 → 6.65	Subjective outcome assessment; limited generalizability	[59]
Single‐blinded crossover	12 adults	Garlic 2000 mg post‐exercise	3 h recovery	Muscle glycogen & mitochondrial markers	↑ glycogen, PGC‐1α (*p* < 0.05), GLUT4 NS	Acute intervention; mechanistic outcomes only	[60]
Multiple RCTs	Men with mild hypertension, hypercholesterolemia, atherosclerosis	Garlic powder	8–52 weeks	Cardiovascular markers	↓ SBP 5.2%, ↓ DBP 4%, LDL↓11.8%, HDL↑11.5%; ↓ platelet aggregation 25.4%	Heterogeneity in garlic preparations and trial designs	[61]
RCT double‐blind placebo	42 men, mild hypercholesterolemia	Time‐released garlic 600 mg/day	12 weeks	Lipid profile	TC↓7.6%, LDL↓11.8%, HDL↑11.5%	Small male‐only sample; short duration	[62]
RCT double‐blind	55 with mild hypercholesterolemia	Aged black garlic 6 g/day	12 weeks	Lipids	HDL↑, ApoB↓, LDL/ApoB improved; TG & LDL NS	Specific aged garlic formulation limits comparison with other garlic products.	[63]
RCT 8‐week	97 women with PCOS & MetS	Garlic 500 mgBID	8 weeks	Metabolic & inflammatory markers	FBS↓10.5%, TG↓17.8%, LDL↓14.2%, TC↓12.6%, CRP↓24.7%	Short duration; limited clinical outcomes	[64]
RCT double‐blind	88 chronic hepatitis patients	Garlic oil + DDB 25/50 mg	6 weeks	Hepatoprotective	ALT normalized: 16%→88% in dose groups; AST ↓	Short duration and small sample size	[65]
RCT double‐blind	58 TB patients	Garlic tablets 1000 mg/day	2 weeks	Prevent hepatotoxicity	13% drug‐induced hepatotoxicity; no significant effect (*p* = 1.0)	Small sample and short follow‐up	[66]
Double‐blind crossover	14 healthy volunteers	Garlic oil 9.9 g	Single dose	Platelet aggregation	Adrenaline‐induced aggregation ↓ 12%, collagen & ADP NS	Very small sample and single‐dose exposure	[67]
RCT double‐blind	44 postmenopausal osteoporotic women	Garlic 2 tablets/day	1 month	Cytokines	TNF‐α ↓ 31.14 → 19.33 ng/mL (≈47%, *p* = 0.05); IL‐1, IL‐6 NS	Small sample and short intervention period	[68]

**TABLE 4:** Preclinical/animal/in vitro studies of garlic.

Study design	Model/sample size	Dose	Duration	Mainoutcomes/effects	Findings	References
In vivo murine experiment	Mice	Garlic oil‐loaded liposomes	Single LPS‐induced ALI	Lung injury, oxidative stress	↓ Pulmonary edema, improved histopathology, Nrf2 pathway activation	[69]
Therapeutic trial	Nile tilapia, 120 fish	Garlic & onion extracts, 1/10 & 1/5 LC50	3 days	Hematology, immune cytokines, gill histology	Restoration of IL‐1β, TNF‐α; improved HB, MCH, WBCs; epithelial regeneration	[70]
Preclinical experimental	60 BALB/c mice	Garlic extracts (fresh, microwaved, etc.)	3 weeks	Tumor inhibition	Tumor size ↓ in all garlic groups (*p* < 0.05); fresh garlic most effective	[71]
Experimental	Mice infected with *Plasmodium* *berghei*	α, β‐arteether + garlic	Single dose	Antimalarial, NO pathway	~95% protection, ↑ NO, ↑ iNOS mRNA	[72]
Preclinical meta‐analysis	Animal models of DKD	Garlic & components	4–10 weeks	Renal function & metabolism	Improved BUN, Scr, proteinuria; ↓ oxidative stress & inflammation	[73]

### 5.1 Antioxidant and Anti‐Inflammatory Effects

Garlic is recognized for its strong antioxidant and anti‐inflammatory properties [21]. These effects are mainly due to its sulfur‐containing bioactive compounds, including allicin, SAC, diallyl disulfide, and diallyl trisulfide. These compounds neutralize ROS, such as superoxide anions, hydroxyl radicals, and hydrogen peroxide, reducing oxidative damage in cells. Garlic enhances natural antioxidant defenses by increasing SOD, CAT, and glutathione peroxidase (GPx) activity. It also inhibits lipid peroxidation and maintains intracellular glutathione (GSH) levels, preserving redox balance [74]. Supplementation with aged garlic extract decreases plasma MDA levels and improves overall antioxidant capacity in conditions associated with oxidative stress [22].

In addition, garlic exerts strong anti‐inflammatory effects. Its compounds inhibit nuclear factor‐kappa B (NF‐κB), reducing the expression of proinflammatory cytokines including TNF‐α, IL‐1β, and IL‐6. Garlic also downregulates cyclooxygenase‐2 (COX‐2) and inducible nitric oxide synthase (iNOS), limiting prostaglandin‐ and nitric oxide‐mediated inflammation [75]. It modulates macrophage and lymphocyte activity, supporting immune homeostasis. The combined antioxidant and anti‐inflammatory effects of garlic contribute to protective outcomes against cardiovascular disorders, neurodegenerative conditions, diabetes, and cancer. By reducing oxidative stress and chronic inflammation, which are two major drivers of disease, garlic is a promising adjunct in preventive and therapeutic strategies [8].

Hou et al. [69] conducted an in vivo murine experimental study to assess garlic oil‐loaded liposomes (GO‐lips) in LPS‐induced acute lung injury. Mice were divided into four groups (normal control, LPS model, garlic oil, and GO‐lip groups). GO‐lips showed a 175 ± 3 nm particle size, PDI of 0.27 ± 0.02, and 70.74 ± 2.11% encapsulation efficiency. GO‐lips improved lung histology, reduced edema, and decreased oxidative and inflammatory markers through Nrf2 pathway activation. These findings show strong mechanistic evidence; however, human translation requires clinical validation and long‐term safety assessment. Tsao et al. [57] performed a single‐blind crossover clinical study involving 11 healthy males receiving garlic (1000 mg/day for ‍4 weeks) during a 40‐km cycling trial. Garlic increased TAC and reduced MDA, TNF‐α, and LDH (*p* < 0.05). However, it did not improve performance, glucose, NEFA, IL‐6, uric acid, or creatine kinase. The crossover design strengthens reliability, but the small sample size, short duration, and healthy male population limit generalizability.

Dehghani et al. [58] conducted a randomized, double‐blind, placebo‐controlled trial including 80 post‐menopausal overweight/obese women with knee osteoarthritis. Participants received garlic (1000 mg/day) or placebo for 12 weeks. Garlic reduced resistin (6.41 ± 2.40 to 5.56 ± 2.16 ng/mL) and pain scores (6.8 ± 2.0 to 5.3 ± 2.0) compared with placebo (6.7 ± 2.4 to 6.2 ± 2.5), while TNF‐α remained unchanged. The randomized design supports evidence quality; however, selective biomarker effects and moderate sample size limit conclusions regarding broader anti‐inflammatory efficacy. Amirsalari et al. [59] conducted a randomized, placebo‐controlled, triple‐blind clinical trial involving 60 women with endometriosis. Participants received garlic tablets (400 mg) plus routine care or placebo for 3 months. Pain scores decreased from 6.51 ± 0.86 to 1.83 ± 1.25 in the garlic group, whereas the control group increased from 6.41 ± 1.12 to 6.65 ± 1.37. Triple blinding and repeated measurements strengthen validity; however, the small sample size and reliance mainly on subjective pain outcomes require confirmation in larger trials.

Cheng et al. [60] conducted a single‐blinded, counterbalanced crossover study involving 12 participants receiving garlic extract (2000 mg) after cycling (60 min at 75% VO_2_max) with a high‐glycemic meal (2 g/kg body weight). Garlic improved muscle glycogen replenishment but did not affect GLUT4 expression or most mitochondrial biogenesis markers. PGC‐1α, TFAM, and FIS1 increased. The crossover design and muscle biopsy assessment provide mechanistic strength. However, the small sample size, short recovery period (3 h), and limited molecular effects restrict conclusions about long‐term metabolic adaptation.

### 5.2 Cardioprotective Effects (Blood Pressure, Lipid Profile, and Endothelial Function)

Garlic has been widely studied for its cardioprotective effects [21]. These benefits are largely due to sulfur‐containing bioactive compounds such as allicin, diallyl sulfides, and S‐allyl cysteine. These compounds have antioxidant, anti‐inflammatory, and vasodilatory properties. Supplementing with garlic can reduce both systolic and diastolic blood pressure, especially in hypertensive patients. This occurs through enhanced nitric oxide availability, which promotes vasodilation, and by inhibiting angiotensin‐converting enzyme (ACE). Meta‐analyses report average reductions of 8–10 mmHg in systolic and 5–6 mmHg in diastolic pressure [8]. Garlic also improves lipid profiles. It lowers total cholesterol, LDL cholesterol, and triglycerides while slightly increasing HDL cholesterol. These effects result from the inhibition of hepatic cholesterol synthesis through downregulation of HMG‐CoA reductase. Antioxidant activity further reduces LDL oxidation and atherogenesis [22].

Karagodin et al. [61] reported that garlic reduced blood pressure (SBP −5.2%, DBP −4.0%), improved lipid profiles (LDL −11.8%, HDL +11.5%), reduced platelet aggregation (25.4%), and lowered 10‐year coronary risk (13.2% in men; 7.1% in women). This study supports cardioprotective effects; however, differences in garlic formulations and study populations limit generalizability. Peleg et al. [76] performed a 16‐week double‐blind, placebo‐controlled study in 33 patients with primary hypercholesterolemia receiving garlic containing 22.4 mg/day of alliin. No significant changes were observed in total cholesterol, LDL, HDL, triglycerides, or psychological outcomes. The negative findings highlight variability related to formulation, dose, and patient characteristics. Jung et al. [63] conducted a 12‐week double‐blind randomized controlled trial involving 55 participants with mild hypercholesterolemia receiving aged black garlic (6 g/day). LDL, triglycerides, total cholesterol, and free fatty acids were unchanged, but HDL increased, apolipoprotein B decreased, and the LDL/ApoB ratio improved. The study suggests selective cardioprotective effects; however, limited sample size and lack of major lipid reductions require further validation.

### 5.3 Antimicrobial and Antiviral Activities

Garlic has broad antimicrobial and antiviral effects [20]. These properties are mainly due to sulfur‐containing compounds such as allicin, diallyl sulfides, S‐allyl cysteine, and ajoene. These bioactive molecules act against bacteria, fungi, and viruses, making garlic a promising natural therapeutic agent. Its antibacterial activity affects both Gram‐positive and Gram‐negative bacteria. Allicin reacts with thiol groups in bacterial enzymes, disrupting key metabolic processes and leading to cell death [21]. Studies show effectiveness against pathogens such as Escherichia coli, *Staphylococcus aureus* (including MRSA), Salmonella spp., *Helicobacter pylori*, and *Pseudomonas aeruginosa*. Some studies demonstrate that garlic extracts can reduce bacterial growth and biofilm formation [77]. These support its use as an adjunct to conventional antibiotics.

Garlic also exhibits antifungal effects. It acts against *Candida* species, *Aspergillus*, and dermatophytes. Allicin and related sulfur compounds disrupt fungal cell membranes and inhibit essential enzymes. In *Candida albicans*, garlic reduces epithelial adherence and prevents hyphal formation [56]. Additionally, garlic shows antiviral activity against both DNA and RNA viruses. This includes influenza, herpes simplex virus (HSV‐1 and HSV‐2), human cytomegalovirus, rhinovirus, and coronaviruses. Allicin interferes with viral replication [56]. It also stimulates immunity by enhancing natural killer (NK) cell activity and macrophage function. Mechanistically, garlic bioactives work through protein thiolation, membrane disruption in bacteria and fungi, and immunomodulation via cytokine production, NK cell activation, and macrophage stimulation [56]. Regular garlic consumption may lower the frequency and severity of colds and respiratory infections. Garlic supplementation has also improved *H. pylori* eradication rates when used as an adjunct therapy [56]. Therapeutic outcomes vary depending on the garlic preparation. Raw garlic, aged extracts, oil, and capsules differ in bioactive compound content, which affects efficacy [19, 20].

de Oliveira Alves et al. [78] conducted a systematic review and meta‐analysis of 9 randomized clinical trials (RCTs) evaluating garlic‐based mouthwashes against cariogenic microorganisms. The studies mainly assessed *Streptococcus mutans*, followed by *Lactobacillus* spp. and *Candida albicans*. Garlic mouthwash significantly reduced *S. mutans* compared with chlorhexidine at 1 week and showed superior activity compared with sodium fluoride at 2 weeks. However, no significant difference was observed at 1 month. The risk of bias was generally low; however, the certainty of evidence was rated low, highlighting the need for further high‐quality trials. Zhang et al. [79] performed a long‐term cohort study assessing garlic and micronutrient supplementation in a rural Chinese population. Among 3411 participants, 34 subjects per treatment stratum were analyzed for total cholesterol, HDL, and LDL at 3.3 and 7.3 years. Long‐term garlic supplementation did not significantly affect lipid profiles. This suggests limited long‐term effects of garlic on cholesterol regulation in this population.

Mohammadzadeh et al. [80] conducted a randomized clinical trial involving 120 women aged 18–44 years comparing garlic tablets (Garsin) with oral metronidazole for bacterial vaginosis. Participants received treatment for 7 days. Both garlic and metronidazole significantly reduced Amsel’s criteria scores, with improvement rates of 63.3% and 48.3%, respectively. Garlic was associated with fewer adverse effects, suggesting potential as an alternative therapy. Rouf et al. [81] performed a systematic review of preclinical and clinical evidence on the antiviral effects of garlic and its organosulfur compounds. Proposed mechanisms included inhibition of viral entry, suppression of viral replication pathways, and immune modulation. However, clinical evidence remains limited and requires further validation. Taghavi et al. [82] conducted a randomized controlled trial evaluating fortified garlic extract (Gallecina) as adjunctive therapy in noncritically ill COVID‐19 patients. Participants received remdesivir plus garlic capsules or placebo. The garlic group required less supplemental oxygen on days 3 and 4 and at discharge. However, no significant difference was observed in overall clinical status on day 6. Gastrointestinal side effects were slightly more frequent in the garlic group, but the difference was not statistically significant.

### 5.4 Anticancer Properties (Mechanisms and Molecular Targets)

Garlic shows significant anticancer activity [83]. This effect is largely due to its organosulfur compounds (OSCs), including DAS, DADS, DATS, SAC, and ajoene. These compounds act on multiple stages of cancer development, including initiation, promotion, and progression. Garlic bioactives induce apoptosis through both intrinsic and extrinsic pathways. They increase mitochondrial membrane permeability and promote cytochrome c release. Caspases are activated, Fas/FasL signaling is enhanced, Bcl‐2 is downregulated, Bax is upregulated, p53 is activated, and PARP is cleaved [84]. These compounds also inhibit cell proliferation by arresting the cell cycle, especially at the G2/M phase. This occurs through upregulation of p21 and p27, inhibition of CDK1 and CDK2, and modulation of cyclins such as cyclin B1 and cyclin D1 [84].

Garlic suppresses angiogenesis. It reduces VEGF expression and VEGFR2 signaling. Matrix metalloproteinases (MMP‐2 and MMP‐9) are downregulated, which decreases new blood vessel formation. Anti‐metastatic effects are mediated by regulating adhesion molecules (E‐cadherin, integrins), inhibiting MMP activity, and limiting epithelial‐mesenchymal transition (EMT) markers [85]. It also provides chemopreventive benefits. Garlic inhibits phase I enzymes like cytochrome P450s, reducing activation of procarcinogens. At the same time, it induces phase II enzymes, including glutathione S‐transferase and quinone reductase, enhancing detoxification. Its antioxidant and anti‐inflammatory properties decrease reactive oxygen species, reduce lipid peroxidation, and suppress NF‐κB signaling. This leads to lower levels of proinflammatory cytokines such as TNF‐α, IL‐6, and COX‐2 [86]. Emerging evidence shows garlic also affects epigenetic regulation. It inhibits histone deacetylases (HDACs), which activates tumor suppressor genes. It can also restore DNA methylation patterns to suppress oncogene expression [87].

Epidemiological studies link regular garlic intake with lower risks of gastrointestinal cancers, including gastric, colorectal, and esophageal cancers. Some studies indicate that garlic, when used as an adjunct therapy, can reduce tumor markers and improve chemotherapy outcomes. However, high‐quality randomized controlled trials remain limited [20]. Garlic acts through multiple mechanisms. These include apoptosis induction, cell cycle arrest, angiogenesis inhibition, metastasis suppression, modulation of carcinogen metabolism, antioxidant and anti‐inflammatory effects, and epigenetic regulation. These properties make garlic a promising candidate for chemoprevention and as an adjunct in cancer therapy [83].

Pourreza et al. [88] conducted a systematic review evaluating garlic in nonalcoholic fatty liver disease (NAFLD). The evidence suggested that garlic may reduce NAFLD progression by improving body weight, lipid and glucose metabolism, and reducing inflammation and oxidative stress. However, limited human clinical trials, heterogeneity in garlic preparations and study designs, and possible publication bias restrict definitive conclusions. Shirzad et al. [71] conducted a randomized preclinical experimental study in 60 BALB/c mice with WEHI‐164 tumor cells. Mice were divided into 6 groups (10 mice/group) and received different garlic extracts (20 mg/kg/0.2 mL intraperitoneally) or saline control for 3 weeks. All garlic‐treated groups showed significant tumor size reduction, with fresh and microwaved garlic producing the strongest effects. Anticancer activity correlated with higher levels of allicin, flavonoids, and phenolic compounds. However, limited sample size, short duration, nonoral administration, and lack of toxicity evaluation restrict translation to humans.

### 5.5 Metabolic Effects (Anti‐Diabetic and Anti‐Obesity)

Garlic exhibits significant metabolic benefits, particularly for diabetes and obesity [8, 22]. Both preclinical and clinical studies consistently demonstrate these antioxidant, anti‐inflammatory, and cytoprotective effects of garlic. Its sulfur‐containing compounds, including allicin, SAC, and diallyl disulfide, mediate most of its actions. Garlic increases insulin secretion from pancreatic β‐cells and improves insulin sensitivity in peripheral tissues. It also lowers hepatic glucose production by modulating gluconeogenic enzymes, such as glucose‐6‐phosphatase and phosphoenolpyruvate carboxykinase, which enhances glycemic control [22]. Additionally, garlic has antioxidant properties that reduce oxidative stress, a key contributor to β‐cell dysfunction. It modulates lipid metabolism by lowering serum triglycerides and LDL cholesterol while increasing HDL cholesterol, indirectly supporting glucose regulation [89]. Garlic supplementation (300–1200 mg/day of standardized extract) significantly reduces fasting blood glucose and HbA1c in type 2 diabetes patients [89].

Regarding anti‐obesity effects, garlic suppresses adipogenic transcription factors such as PPARγ and C/EBPα, limiting the differentiation of fat cells. It also enhances energy expenditure by upregulating uncoupling proteins and modulates lipid metabolism to reduce visceral fat [89]. Garlic may influence satiety hormones, including leptin and ghrelin, and can alter gut microbiota composition, further supporting weight management [21, 90]. The combined anti‐diabetic and anti‐obesity actions make garlic a promising adjunct therapy for metabolic syndrome. It simultaneously improves glycemic control, oxidative stress, lipid metabolism, and fat accumulation [8].

Fu et al. [91] conducted a meta‐analysis of 19 RCTs involving 999 participants. Garlic supplementation significantly reduced triglycerides, total cholesterol, LDL‐C, BMI, waist circumference, and diastolic blood pressure. Meta‐regression identified age and sample size as potential effect modifiers. However, heterogeneity and possible publication bias indicate the need for larger, high‐quality trials. Zhao et al. [92] performed a systematic review and meta‐analysis of RCTs evaluating garlic effects on glucose and lipid metabolism. Garlic reduced fasting blood glucose by 7.01 mg/dL, HbA1c by 0.66%, total cholesterol by 14.17 mg/dL, and LDL‐C by 8.20 mg/dL. It also increased HDL‐C by 2.06 mg/dL. No significant effect was observed on triglycerides, suggesting variability among studies.

Hesari et al. [64] conducted an 8‐week RCT in 97 women with PCOS and metabolic syndrome. Garlic tablets (500 mg containing 2–3 mg allicin, twice daily) significantly improved fasting blood glucose, triglycerides, LDL‐C, total cholesterol, CRP, SHBG, quality of life, and sexual function. Blood pressure, weight, and BMI also improved. However, the small sample size and short duration limit long‐term conclusions. Sangouni et al. [93] conducted a 3‐month RCT involving 90 adults with metabolic syndrome receiving garlic powder (1600 mg/day). Garlic increased HDL‐C and reduced waist circumference, systolic and diastolic blood pressure, triglycerides, fasting insulin, HOMA‐IR, fatty liver index, and appetite‐related scores. The limited sample size and short follow‐up reduce generalizability. Sangouni et ‍al. [94] performed a 12‐week RCT in 90 patients with nonalcoholic fatty liver disease (NAFLD) using garlic powder (4 × 400 mg/day). Garlic significantly reduced waist circumference, body fat percentage, fasting blood glucose, insulin, HOMA‐IR, and malondialdehyde. It also increased skeletal muscle mass, superoxide dismutase activity, and total antioxidant capacity. Despite promising metabolic effects, the short duration and disease‐specific sample limit broader clinical interpretation.

### 5.6 Hepatoprotective Effects

Garlic protects the liver against damage caused by toxins, drugs, oxidative stress, and metabolic disturbances [95]. Its protective effects are mainly due to organosulfur compounds such as allicin, SAC, and diallyl sulfides. It also contains flavonoids and phenolic compounds, which have strong antioxidant and anti‐inflammatory properties [6]. Garlic boosts the liver’s natural antioxidant defenses. It increases the activity of SOD, CAT, and glutathione peroxidase (GPx). At the same time, it scavenges ROS, which reduces oxidative stress in liver cells [6]. It also reduces inflammation in the liver. Garlic suppresses proinflammatory cytokines, including TNF‐α, IL‐1β, and IL‐6. It downregulates NF‐κB signaling, which helps prevent inflammation‐related liver injury [6].

Garlic supports liver detoxification. It stimulates phase II detoxifying enzymes, such as glutathione S‐transferase, and modulates cytochrome P450 enzymes [96]. These effects help metabolize and remove hepatotoxic substances like acetaminophen and carbon tetrachloride (CCl_4_). Additionally, garlic has anti‐fibrotic effects. It inhibits hepatic stellate cell activation, reduces collagen buildup, and prevents extracellular matrix accumulation. This helps stop the progression of liver fibrosis [96]. Garlic protects the liver through antioxidant, anti‐inflammatory, detoxifying, and anti‐fibrotic mechanisms. Its organosulfur compounds, including allicin and S‐allyl cysteine, play central roles in these protective actions [88].

Lee et al. [65] conducted a randomized, double‐blind, placebo‐controlled trial in 88 patients with histologically confirmed chronic hepatitis to evaluate DDB (25 mg) plus garlic oil (50 mg) capsules. Of 83 treated patients, 79 completed the study, and 81/83 (98%) were HBV‐positive. At week 6, ALT normalization was 16% in the placebo group, 41% in the low‐dose group, 52% in the medium‐dose group, and 88% in the high‐dose group, showing a dose‐dependent hepatoprotective effect. No clinically significant adverse events were reported. The randomized design, dose–response evaluation, and biochemical outcomes strengthen the evidence. However, short follow‐up, moderate sample size, HBV‐dominant population, and lack of long‐term clinical outcomes limit generalizability.

Tabarsi et al. [66] conducted a randomized, double‐blind, placebo‐controlled trial in 58 newly diagnosed pulmonary tuberculosis patients receiving garlic tablets (1000 mg/day) during the first 2 weeks of anti‐tuberculosis therapy (garlic *n* = 31; placebo *n* = 27). Hepatotoxicity occurred in 8 patients (13%), including 6 cases within the first 2 weeks, with no significant protective effect of garlic. The rigorous design and clinically relevant outcome were strengths. However, small sample size, short intervention duration, few hepatotoxicity events, and limited follow‐up reduced statistical power.

### 5.7 Immunomodulatory Effects

Garlic has immunomodulatory properties involving both innate and adaptive immune responses. Its sulfur‐containing compounds, including allicin, diallyl sulfides, S‐allyl‐L‐cysteine (SAC), and ajoene, together with flavonoids and polysaccharides, contribute to these effects [8]. In innate immunity, garlic promotes the activity of natural killer (NK) cells, macrophages, and dendritic cells. Garlic extracts enhance phagocytosis and stimulate the production of immune mediators, including IL‐1β, TNF‐α, and IFN‐γ, thereby supporting host defense against pathogens [20]. Garlic also influences adaptive immunity by affecting T and B lymphocytes. It promotes T‐helper cell proliferation, enhances antibody production, and modulates the Th1/Th2 balance. These effects may contribute to effective immune responses and antiviral defense [20]. In conditions characterized by excessive immune activation, SAC and allicin can modulate NF‐κB signaling and reduce excessive proinflammatory cytokine production [97].

Additional mechanisms include modulation of MAPK and JAK/STAT signaling, antioxidant protection of immune cells, and epigenetic regulation of cytokine‐related genes. Interactions among garlic constituents may further contribute to its overall immunomodulatory activity [22]. Further research is required to establish optimal doses, treatment duration, and standardized preparations for clinical application [85]. Vathsala et al. [72] conducted an experimental in vivo study in *Plasmodium* *berghei*‐infected mice. A single dose of α, β‐arteether combined with three oral doses of garlic provided approximately 95% protection, with increased nitric oxide (NO), iNOS expression, and T‐cell responses, resulting in reduced parasitemia and improved survival. The study provides strong mechanistic evidence; however, limited human applicability, small experimental scale, and short‐term evaluation restrict clinical translation. Mozaffari‐Khosravi et al. [68] conducted a double‐blind randomized controlled trial involving 44 postmenopausal osteoporotic women treated with garlic tablets for 1 month. Garlic significantly reduced TNF‐α by approximately 47% (31.14 ± 50.53 to 19.33 ± 22.19 ng/ml), while IL‐1 and IL‐6 showed no significant changes. The randomized placebo‐controlled design strengthens the findings, but the small sample size, short duration, and limited cytokine effects reduce generalizability.

### 5.8 Anti‐Thrombotic/Platelet Aggregation Effects

Garlic exhibits antithrombotic and antiplatelet activity through direct effects on platelet activation and aggregation. Its organosulfur compounds, including allicin, diallyl disulfide (DADS), diallyl trisulfide (DATS), and ajoene, can interfere with platelet responses to agonists such as ADP, collagen, and epinephrine. These effects are associated with modulation of arachidonic acid metabolism and platelet‐specific signaling pathways [20, 98]. Garlic‐derived sulfur compounds may inhibit cyclooxygenase‐dependent thromboxane A_2_ formation, thereby reducing platelet activation and subsequent aggregation. They may also interfere with intracellular calcium mobilization, which regulates platelet shape change, granule secretion, and aggregation. In addition, garlic constituents may enhance nitric oxide (NO)‐dependent signaling and cyclic guanosine monophosphate (cGMP) activity, which contribute to platelet inhibition [20]. Garlic constituents may further inhibit glycoprotein IIb/IIIa (GPIIb/IIIa) activation, thereby reducing fibrinogen‐mediated platelet–platelet bridging. Ajoene has been particularly investigated for its ability to inhibit platelet activation and aggregation. The antiplatelet effects of garlic may therefore result from combined interference with thromboxane A_2_ production, calcium‐dependent platelet signaling, NO/cGMP pathways, and GPIIb/IIIa‐mediated platelet aggregation. However, the magnitude of these effects may vary according to the garlic preparation, constituent composition, dose, duration of exposure, and experimental conditions.

Mollahosseini et al. [98] conducted a systematic review of RCTs on garlic and platelet aggregation. Among 18,235 identified articles, 12 RCTs involving 595 participants were included. Six studies reported significant reductions in platelet aggregation. However, heterogeneity and small sample sizes limited definitive conclusions, indicating the need for larger high‐quality trials. Wojcikowski et al. [67] performed a double‐blind, placebo‐controlled crossover study in 14 healthy volunteers using garlic oil derived from 9.9 g of garlic. Garlic oil produced only a 12% reduction in adrenaline‐induced platelet aggregation at 4 h, with no significant effects on collagen‐ or ADP‐induced aggregation. The findings suggest limited antiplatelet activity of this specific preparation. However, interpretation is restricted by the small sample size and formulation‐specific effects. Fakhar and Hashemi Tayer [99] conducted a randomized clinical trial involving 36 healthy volunteers comparing garlic tablets (600, 1200, and 2400 mg/day) with 75 mg Plavix for 3 weeks. Garlic at 1200 and 2400 mg/day significantly reduced ADP‐ and arachidonic acid‐induced platelet aggregation, while 2400 mg/day increased bleeding time. The study demonstrates dose‐dependent antiplatelet effects. However, it is limited by short duration, small sample size, and inclusion of only healthy participants.

### 5.9 Renoprotective Effects

Garlic protects the kidneys. Its bioactive sulfur compounds, including allicin, S‐allyl cysteine (SAC), diallyl sulfides, and polyphenols, provide antioxidant, anti‐inflammatory, and anti‐fibrotic effects [21]. It strengthens antioxidant defenses. Garlic increases superoxide dismutase, catalase, and glutathione peroxidase. At the same time, it reduces reactive oxygen species and lipid peroxidation [8]. These actions protect kidney cells from oxidative damage, which is a key factor in nephrotoxicity and kidney dysfunction. Garlic also reduces inflammation in the kidneys. It lowers proinflammatory cytokines such as TNF‐α and IL‐6. It inhibits NF‐κB signaling, helping to prevent inflammatory damage [8]. Kidney blood flow improves with garlic. It enhances nitric oxide availability, supporting renal perfusion and glomerular filtration [22]. Anti‐fibrotic effects occur through suppression of fibrotic markers like TGF‐β1 and collagen deposition. This prevents kidney fibrosis, a hallmark of chronic kidney disease.

Garlic protects against nephrotoxicity from heavy metals, such as cadmium and lead, and from drugs like gentamicin and cisplatin. It activates detoxification pathways and scavenges free radicals [95]. It preserves kidney function by reducing markers of injury and maintaining kidney structure. Garlic also prevents glomerular hypertrophy, mesangial expansion, and proteinuria, helping to limit progressive kidney damage [75]. Garlic is generally safe at dietary doses. High doses of concentrated extracts may interact with anticoagulants, antihypertensives, or antiplatelet drugs [56]. This is especially important for people with kidney impairment. Garlic protects the kidneys through antioxidant, anti‐inflammatory, and anti‐fibrotic mechanisms. It may serve as an adjunct therapy for kidney health [100].

Jiang et al. [73] conducted a preclinical systematic review and meta‐analysis of 24 animal trials from 8 databases to evaluate garlic’s renoprotective effects in diabetic kidney disease. Garlic improved BUN, serum creatinine, proteinuria, 24‐h urine volume, kidney index, blood glucose, insulin, and body weight. Effects were linked to reduced oxidative stress, inflammation, and renal fibrosis. Dose–time analysis suggested optimal effects with garlic at 500 mg/kg for 8–10 weeks or garlic components at 45–150 mg/kg for 4–10 weeks. The study provides a comprehensive synthesis and mechanistic evaluation; however, the available evidence is primarily derived from animal models. Furthermore, heterogeneity among studies, variations in study quality, potential risk of bias, and limited translation of findings to human clinical settings should be considered.

### 5.10 Anti‐Parasitic Effects

Garlic has strong anti‐parasitic activity against a variety of protozoa, helminths, and ectoparasites. Its effects are mainly due to sulfur‐containing compounds such as allicin, diallyl sulfide, diallyl disulfide, and ajoene [101]. These compounds act through multiple mechanisms. They interfere with parasite metabolism by reacting with thiol groups in essential enzymes. They also induce oxidative stress, which damages membranes, proteins, and nucleic acids. Garlic reduces parasite reproduction and growth by lowering egg production, cyst formation, or trophozoite proliferation. It can also disrupt membrane integrity through interactions with lipids and proteins [102]. Garlic is effective against protozoa such as Giardia lamblia, Entamoeba histolytica, and Plasmodium species. It also acts against nematodes like Ascaris lumbricoides and Haemonchus contortus [70]. Its use has been associated with reductions in parasite load and improved host responses, supporting its potential as a natural anthelmintic [101]. Some reports suggest it can reduce parasite burden in humans [70].

Reda et al. [70] conducted a randomized controlled therapeutic trial in 120 Nile tilapia (Oreochromis niloticus) infected with Dactylogyrus sp. to evaluate garlic extract (GE) and onion extract (OE). Fish were allocated into 6 groups (30 fish/group; 10 per replicate) and treated for 3 days with 1/10 and 1/5 LC50 doses. The 96‐h LC50 was 0.4 g/L for GE and 3.54 g/L for OE, with in vitro antiparasitic activity of 0.02–0.18 mg/mL for GE and 0.4–1.8 mg/mL for OE. GE and OE improved hematological parameters, innate immunity markers, cytokine expression (IL‐1β and TNF‐α), and gill histopathology. The study had strengths, including a randomized design, dose evaluation, and combined in vitro–in vivo assessment. However, the short duration, lack of pharmacokinetic and long‐term safety data, incomplete parasite burden evaluation, and fish model limit generalizability.

### 5.11 Comparative Summary of Major Randomized Controlled Trials Evaluating the Clinical Effects of Garlic

Clinical investigations have evaluated garlic supplementation across diverse populations, including individuals with metabolic disorders, cardiovascular risk factors, inflammatory conditions, and chronic diseases. RCT evidence suggests that garlic may exert beneficial effects on lipid metabolism, blood pressure, inflammatory biomarkers, oxidative stress, and symptom severity. However, differences in garlic preparation (fresh garlic, aged garlic extract, garlic powder, garlic oil, and time‐released formulations), dosage, intervention duration, and participant characteristics contribute to variability among studies. Importantly, while several trials demonstrate statistically significant improvements in surrogate biomarkers, evidence for long‐term clinical outcomes remains limited.

## 6 Mechanistic Insights of Garlic

Garlic exerts its biological activities through a coordinated multitarget molecular network involving tightly interconnected redox, inflammatory, metabolic, mitochondrial, immune, and antimicrobial pathways [69] (Figure 2). The following integrated framework organizes all mechanisms without redundancy while preserving full conceptual coverage.

**FIGURE 2:**
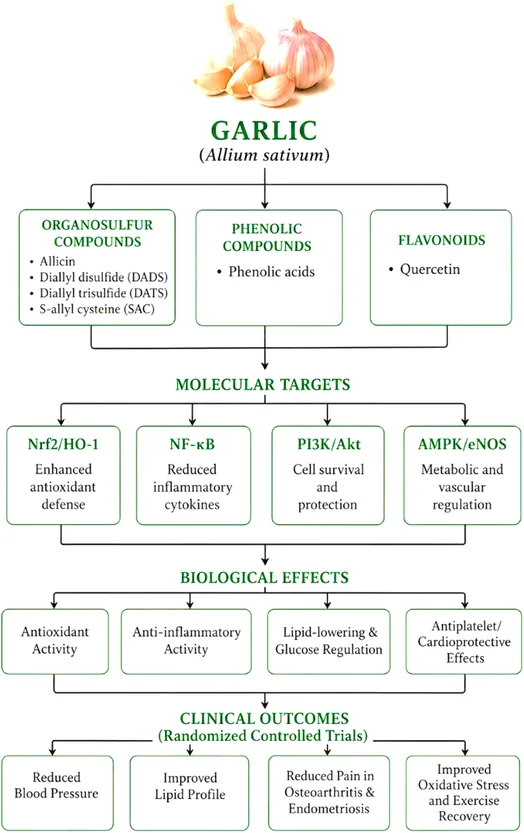
Bioactive constituents, molecular targets, biological effects, and clinical outcomes of garlic.

### 6.1 Redox Homeostasis and Antioxidant Signaling (Nrf2–ARE Axis)

Garlic primarily regulates cellular redox balance through activation of the Nrf2–ARE signaling pathway, which serves as a master regulator of antioxidant defense systems [57, 69]. Bioactive sulfur compounds promote Nrf2 nuclear translocation, leading to transcriptional activation of endogenous antioxidant enzymes, including superoxide dismutase, catalase, and glutathione peroxidase [57, 69, 73]. Nrf2 activation can also increase the expression of heme oxygenase‐1 (HO‐1), an important cytoprotective enzyme involved in antioxidant and cellular stress responses. Garlic‐derived organosulfur compounds, including diallyl sulfide, diallyl disulfide, diallyl trisulfide, and S‐allyl cysteine, have been reported to promote Nrf2/HO‐1 signaling, thereby strengthening endogenous antioxidant defenses [56]. This reduces intracellular reactive oxygen species accumulation and limits lipid peroxidation–driven cellular injury. Experimental evidence indicates that garlic‐derived formulations enhance Nrf2‐dependent cytoprotective signaling in oxidative stress and inflammatory injury models [57, 69, 73].

### 6.2 Inflammatory Signal Regulation (NF‐κB and Cytokine Axis)

Garlic regulates inflammatory homeostasis through inhibition of the NF‐κB signaling pathway, a central transcriptional regulator of proinflammatory gene expression [58, 64, 68]. Sulfur‐containing bioactives interfere with upstream kinase activation, preventing NF‐κB p65 nuclear translocation and suppressing transcription of TNF‐α, IL‐1β, IL‐6, and CRP‐associated inflammatory networks [64, 70]. This also extends to modulation of adipokine‐related inflammatory signaling, including resistin‐mediated pathways, contributing to immune–metabolic regulation and attenuation of chronic inflammatory activation [58, 64, 68, 70].

### 6.3 Lipid Metabolism and Vascular Regulation (HMG‐CoA + PPAR + Platelet Axis)

Garlic regulates lipid and cardiovascular homeostasis through multiple interconnected metabolic targets [61, 62]. It inhibits HMG‐CoA reductase, reducing endogenous cholesterol biosynthesis at a rate‐limiting step. In parallel, garlic modulates PPAR nuclear receptor signaling, which governs fatty acid oxidation, lipid transport, and systemic metabolic adaptation. Additionally, garlic influences vascular homeostasis by modulating platelet activation pathways and thromboxane‐related enzymatic signaling, contributing to regulation of endothelial function and hemostatic balance [61, 62, 63]. Garlic‐derived sulfur compounds also influence endothelial nitric oxide signaling through the AMPK/eNOS/NO pathway, supporting NO bioavailability and vascular function. In particular, S‐1‐propenylcysteine, a characteristic sulfur compound of aged garlic extract, has been shown to increase AMPK and eNOS phosphorylation and plasma NO metabolites in experimental models [103].

### 6.4 Glucose and Insulin Signaling Modulation (Redox‐Sensitive Metabolic Pathways)

Garlic influences glucose homeostasis through modulation of insulin receptor signaling pathways and attenuation of oxidative stress–induced insulin resistance [64, 92]. By reducing ROS‐mediated disruption of insulin receptor substrate signaling, garlic enhances intracellular metabolic signaling efficiency. The PI3K/Akt pathway represents an additional signaling component through which garlic‐derived bioactives may influence cellular survival and metabolic regulation. Allicin and other garlic‐derived organosulfur compounds have been reported to modulate PI3K/Akt signaling in experimental models, providing an additional mechanistic link within cellular metabolic signaling [48]. It also interacts with downstream metabolic regulatory networks controlling glucose utilization and energy balance. However, effects on glucose transporter regulation (e.g., GLUT4) appear context‐dependent, suggesting indirect modulation rather than direct transporter activation [60, 64, 92].

### 6.5 Mitochondrial Function and Energy Biogenesis (PGC‐1α Pathway)

Garlic regulates mitochondrial dynamics through activation of the PGC‐1α signaling pathway, a master regulator of mitochondrial biogenesis and oxidative phosphorylation capacity [60]. This pathway coordinates transcriptional programs responsible for mitochondrial replication, metabolic adaptation, and energy efficiency. Garlic bioactives also support mitochondrial structural and functional integrity, thereby maintaining respiratory chain activity and cellular energy production [60].

### 6.6 Immune Effector Activation (iNOS/NO Pathway)

Garlic enhances innate immune defense through activation of the iNOS pathway, leading to increased nitric oxide production. NO functions as both an antimicrobial effector molecule and a regulator of immune cell signaling. It contributes to pathogen clearance and immune surveillance (Vathsala et al. 2020). This pathway therefore provides an immune‐specific mechanism of host defense distinct from the endothelial AMPK/eNOS/NO pathway.

### 6.7 Antimicrobial and Anti‐Biofilm Activity (Thiol‐Reactive Sulfur Chemistry)

Garlic exerts antimicrobial effects through thiol‐reactive organosulfur compounds, particularly allicin [78]. These molecules covalently modify bacterial protein thiol groups, leading to enzyme inactivation and disruption of essential metabolic processes. In addition, garlic interferes with bacterial membrane integrity and inhibits quorum sensing–regulated biofilm formation, thereby impairing microbial adhesion, colonization, and community organization [78]. This chemical reactivity underlies its broad‐spectrum antimicrobial properties.

### 6.8 Integrated Organ‐Level Cytoprotection (Convergent Pathway Model)

At the organ level, garlic mediates cytoprotection through convergence of redox, inflammatory, and mitochondrial signaling networks [65, 66]. The combined activity of these pathways translates into tissue‐specific protection and functional preservation across multiple organ systems. In hepatic systems, these mechanisms regulate detoxification enzyme activity and maintain membrane stability via redox‐sensitive pathways. In renal systems, garlic modulates oxidative stress–driven fibrotic and inflammatory signaling cascades involved in tissue remodeling. In pulmonary systems, these integrated responses contribute to epithelial protection and attenuation of lung injury [69]. In neoplastic systems, garlic interferes with proliferative and oxidative stress–dependent survival signaling pathways, disrupting abnormal cellular growth regulation [65, 66, 69, 70, 71, 73].

Figure 2 illustrates how garlic phytochemicals, including organosulfur compounds, phenolics, and flavonoids, regulate key signaling pathways to produce antioxidant, anti‐inflammatory, metabolic, and cardioprotective effects. These mechanisms are supported by clinical trial evidence showing improvements in blood pressure, lipid profiles, pain severity, oxidative stress status, and exercise recovery.

## 7 Safety and Toxicology

Garlic is generally safe when eaten in normal dietary amounts. Higher doses, especially from concentrated extracts or supplements, can cause side effects [95]. The most common issues involve the digestive system. These include heartburn, bloating, flatulence, nausea, and diarrhea. Such effects are dose‐dependent and occur more often with raw garlic or concentrated preparations [6]. Garlic can also cause breath and body odor due to sulfur compounds like allicin. Rarely, some individuals experience allergic reactions. These may include contact dermatitis or respiratory symptoms [104]. High doses of garlic can reduce platelet aggregation. This may increase bleeding risk, particularly in patients on anticoagulants [105]. Garlic may also interact with medications by affecting cytochrome P450 enzymes and platelet function [106]. Key interactions include increased bleeding risk with drugs such as warfarin, aspirin, and clopidogrel. Garlic may also enhance the blood pressure‐lowering effects of ACE inhibitors or beta‐blockers. In addition, it can amplify the glucose‐lowering effect of insulin or oral hypoglycemics, which requires close monitoring [107]. While garlic may modestly influence drug metabolism, serious interactions are uncommon [108].

Recommended doses vary by preparation. One to two cloves of raw garlic per day (about 3–5 g) is typical. Garlic powder or extract supplements usually provide 300–1000 mg/day of powder or 4–10 mg/day of allicin equivalents. Aged garlic extract (AGE) is often given at 600–1200 mg/day. It is generally well tolerated and produces less odor. Oil macerates or enteric‐coated forms reduce gastrointestinal irritation and allow controlled release for better compliance [17, 22]. Safety measures are important. Avoid high‐dose supplements before surgery or with anticoagulants. Pregnant or breastfeeding women should consume garlic primarily as part of their diet. Start with low doses and monitor tolerance, especially for digestive symptoms [109]. Garlic is safe at culinary doses. Mild gastrointestinal discomfort and odor are the most common side effects. Clinicians should consider potential bleeding or drug interactions when higher doses or supplements are used [110].

Lin et al. [111] evaluated the genotoxicity and 28‐day oral toxicity of garlic essential oil (GEO) in mice using Ames tests with five *Salmonella typhimurium* strains (TA98, TA100, TA102, TA1535, and TA1537), CHO–K1 cells, and micronucleus assays. GEO showed no mutagenic or clastogenic effects. Oral administration of 15, 25, and 50 mg/kg/day for 28 days caused no adverse effects in behavior, survival, body weight, food intake, hematology, serum biochemistry, or organ weights, with a NOAEL > 50 mg/kg/day. Yamato et al. [112] assessed aged garlic extract (AGE) safety in beagles receiving 45 or 90 mg/kg/day for 12 weeks. No clinical, hematological, or biochemical abnormalities were observed, and no Heinz bodies or eccentrocytes were detected. AGE at 90 mg/kg/day increased NFE2L2 (Nrf2), NQO1, and GCLM expression, suggesting safety and enhanced antioxidant defense. Both studies provide strong safety evidence through comprehensive toxicological assessments. However, limitations include short exposure durations (28 days for GEO and 12 weeks for AGE), small sample sizes, and animal models, which limit direct human translation. Long‐term human studies are required to confirm safety, optimal dosing, and potential interactions.

## 8 Discussion

### 8.1 Integration of Clinical and Preclinical Evidence

The current body of evidence indicates that *Allium sativum* L. exerts a broad spectrum of pharmacological effects supported by both experimental and clinical investigations, although the strength of evidence varies substantially across study designs. The most convincing clinical evidence relates to cardiovascular and metabolic disorders, where randomized controlled trials (RCTs), systematic reviews, and meta‐analyses consistently demonstrate improvements in blood pressure, serum lipid profiles, glycemic control, oxidative stress, and selected inflammatory biomarkers [61, 91, 92]. Clinical trials further demonstrate that garlic supplementation significantly reduces pain severity in women with endometriosis, improves symptoms of knee osteoarthritis, enhances antioxidant capacity following exercise, and improves metabolic parameters among women with polycystic ovary syndrome (PCOS). These findings suggest therapeutic potential beyond cardiovascular disease [57, 60, 64].

Preclinical investigations provide important biological support for these clinical observations. Experimental studies consistently demonstrate antioxidant, anti‐inflammatory, nephroprotective, hepatoprotective, antimicrobial, antitumor, antiparasitic, and immunomodulatory activities. For example, garlic oil‐loaded liposomes alleviated lipopolysaccharide‐induced acute lung injury by activating the nuclear factor erythroid 2‐related factor 2 (Nrf2) antioxidant pathway [69]. Experimental diabetic kidney disease models also demonstrated improvements in renal function accompanied by reductions in oxidative stress and inflammatory injury [73]. Likewise, fresh garlic extracts significantly inhibited tumor growth in murine cancer models [71]. Garlic enhanced nitric oxide‐mediated antiparasitic activity against *Plasmodium* *berghei* (Vathsala et al. 2020), and garlic‐based mouthwashes exhibited significant antimicrobial effects against Streptococcus mutans in systematic reviews of randomized trials [78].

### 8.2 Consistency and Contradictory Findings Across Studies

Although the majority of investigations report favorable therapeutic effects, important inconsistencies remain. Cardiovascular outcomes appear to be the most reproducible findings. Several randomized trials demonstrated significant reductions in systolic and diastolic blood pressure together with improvements in lipid profiles, including reductions in total cholesterol and low‐density lipoprotein (LDL) cholesterol and increases in high‐density lipoprotein (HDL) cholesterol [61, 62]. These findings are further supported by recent meta‐analyses showing significant improvements in metabolic syndrome components and glycemic indices [91, 92]. In contrast, inflammatory biomarkers demonstrate greater variability. Dehghani et al. [58] observed significant reductions in circulating resistin concentrations among women with knee osteoarthritis but did not detect significant changes in TNF‐α. Similarly, [68] reported a significant reduction in TNF‐α without corresponding changes in interleukin (IL)‐1 or IL‐6 concentrations.

Tsao et al. [57] demonstrated reductions in TNF‐α during exercise recovery but no significant effect on IL‐6. These observations suggest that garlic does not uniformly suppress all inflammatory cytokines but instead selectively modulates specific inflammatory signaling pathways depending on disease characteristics and physiological conditions. Similarly, inconsistent findings have been reported regarding lipid metabolism. Time‐released garlic tablets significantly reduced total cholesterol and LDL cholesterol in patients with mild hypercholesterolemia [62], whereas aged black garlic primarily increased HDL cholesterol and improved apolipoprotein B (ApoB)‐related indices without producing statistically significant reductions in LDL cholesterol or triglycerides [63]. Furthermore, although garlic oil demonstrated hepatoprotective activity in patients with chronic hepatitis [65], supplementation failed to prevent anti‐tuberculosis drug‐induced hepatotoxicity in another randomized clinical trial [66]. These contradictory findings indicate that therapeutic responses are likely influenced by formulation, disease pathology, treatment duration, and study methodology.

### 8.3 Sources of Heterogeneity

Several methodological and biological factors likely explain the heterogeneity observed across published studies. First, garlic preparations differed considerably among investigations, including raw garlic, garlic powder, garlic oil, aged garlic extract, aged black garlic, time‐released tablets, encapsulated preparations, and liposomal formulations [61, 63, 69]. Because these preparations differ substantially in concentrations of allicin, S‐allyl cysteine, diallyl sulfides, ajoene, and other organosulfur compounds, pharmacological effects cannot be assumed to be equivalent across formulations. Second, standardization of active constituents remains inadequate in many studies. Numerous investigations report garlic doses without quantifying biologically active compounds, making reproducibility and dose–response comparisons difficult. Future studies should routinely characterize allicin yield, S‐allyl cysteine concentration, extraction procedures, and stability of garlic preparations to improve comparability among clinical trials.

Third, intervention dose and duration varied substantially, with clinical doses ranging from 400 mg/day to 2000 mg/day and intervention periods extending from a single administration to 12 weeks or longer [59, 60, 62]. Short‐term interventions may adequately detect changes in oxidative stress biomarkers but are unlikely to capture long‐term clinical outcomes, such as atherosclerotic progression or sustained glycemic improvement. Additional sources of heterogeneity include differences in patient populations, disease severity, baseline inflammatory status, age, sex, and concomitant therapies. Clinical studies enrolled healthy volunteers, athletes, patients with metabolic syndrome, hypercholesterolemia, osteoarthritis, endometriosis, chronic hepatitis, tuberculosis, and PCOS, making direct comparisons challenging [58, 64]. Furthermore, many randomized trials involved relatively small sample sizes, increasing the possibility of type II errors and reducing statistical power. In contrast, systematic reviews and meta‐analyses provide more reliable estimates of treatment effects by integrating evidence from multiple studies [91, 92].

### 8.4 Evidence Stratification

The available literature should be interpreted according to the hierarchy of scientific evidence. The strongest evidence originates from systematic reviews and meta‐analyses demonstrating significant improvements in cardiovascular risk factors, lipid metabolism, glycemic control, and metabolic syndrome [91, 92]. High‐quality randomized double‐blind placebo‐controlled trials provide moderate‐to‐high certainty for improvements in hypercholesterolemia, osteoarthritis, endometriosis, and PCOS [58, ‍^59^, ^62^, ^64^]. Smaller crossover studies involving healthy volunteers provide supportive but lower‐level evidence for antioxidant effects and exercise recovery because of limited sample sizes and short intervention periods [57, 60]. Conversely, evidence derived from animal models, in vivo experiments, and in vitro studies should primarily be regarded as mechanistic rather than confirmatory clinical evidence. Although these investigations consistently demonstrate antioxidant, anti‐inflammatory, nephroprotective, hepatoprotective, antimicrobial, and antitumor activities [68, 71, 73]; (Vathsala et al. 2020), translation of these findings into routine clinical practice requires confirmation through adequately powered randomized clinical trials. Therefore, mechanistic evidence provides biological plausibility but should not be interpreted as equivalent to clinically validated therapeutic efficacy.

## 9 Translational and Clinical Challenges: From Biological Activity to Clinical Application

Although the molecular mechanisms underlying the pharmacological activities of garlic are increasingly characterized, translating these findings into clinical practice remains challenging. Biological activity demonstrated in experimental models does not necessarily translate into therapeutic benefit in humans. Clinical application is influenced by the formulation administered, chemical composition, biological disposition, dose, safety, patient acceptability, and regulatory environment. Addressing these factors is essential for converting experimental findings into consistent and clinically applicable interventions.

### 9.1 Standardization of Garlic Preparations

Garlic has been investigated in multiple forms, including raw garlic, fresh garlic, garlic powder, garlic oil, aged garlic extract, aged black garlic, enteric‐coated tablets, time‐released formulations, liposomal preparations, and encapsulated supplements [61, 63, 69]. These preparations differ in processing methods, chemical stability, and concentrations of bioactive constituents. Such differences can substantially influence biological activity and complicate comparisons among products. Findings obtained with one preparation should therefore not automatically be extrapolated to other formulations or commercial products.

### 9.2 Variability in Bioactive Compound Content

Garlic contains numerous bioactive constituents, including alliin, allicin, diallyl sulfides, ajoene, vinyldithiins, S‐allyl cysteine (SAC), and S‐allyl mercaptocysteine (SAMC). Their concentrations vary according to cultivar, geographical origin, cultivation practices, harvesting time, storage conditions, processing techniques, and manufacturing procedures. Allicin is particularly unstable and rapidly undergoes transformation into secondary sulfur‐containing metabolites following garlic crushing or processing. Consequently, products with similar nominal amounts of garlic may provide substantially different chemical exposures and biological effects.

### 9.3 Pharmacokinetics and Bioavailability

Limited understanding of the pharmacokinetic behavior of garlic‐derived compounds represents an important translational challenge. Allicin is chemically unstable, undergoes rapid metabolism, and has limited systemic availability following oral administration. In contrast, aged garlic extract contains more stable water‐soluble organosulfur compounds, including SAC and SAMC, which exhibit different absorption and pharmacokinetic characteristics. Comprehensive human characterization of absorption, distribution, metabolism, elimination, and exposure‐response relationships remains limited. These differences in biological disposition complicate the development of clinically applicable dosing strategies.

### 9.4 Dose Equivalence Among Commercial Formulations

Uncertainty also exists regarding dose equivalence among commercially available garlic products. Clinical trials have administered approximately 400 mg/day to 2000 mg/day using preparations with substantially different phytochemical compositions [59, 60, 62, 64]. Consequently, equivalent doses expressed as total product weight may correspond to substantially different exposures to biologically active compounds. Comparisons based solely on milligram dose may therefore be misleading.

### 9.5 Long‐Term Safety and Adverse Events

Although garlic is generally considered safe as a food and is well tolerated in most clinical studies, the safety profile of prolonged supplementation remains incompletely characterized. Reported adverse events are generally mild and include gastrointestinal discomfort, abdominal bloating, nausea, heartburn, flatulence, garlic odor, and occasional allergic reactions. However, the effects of prolonged high‐dose supplementation, particularly among older adults and patients with multiple comorbidities, require careful clinical consideration.

### 9.6 Potential Herb–Drug Interactions

Potential herb–drug interactions represent an important clinical consideration. Garlic has antiplatelet activity and may increase bleeding risk when administered with anticoagulant or antiplatelet medications, particularly warfarin, aspirin, or clopidogrel [67]. Garlic‐derived constituents may also influence hepatic drug‐metabolizing enzymes and drug transporters, potentially affecting the disposition of concomitantly administered medicines. Although clinically significant interactions appear relatively uncommon, medication use should be carefully considered in patients receiving multiple therapies or undergoing surgical procedures.

### 9.7 Patient Adherence and Acceptability

Long‐term use may be affected by patient acceptability and treatment burden. The characteristic garlic odor, gastrointestinal effects, dosing frequency, and pill burden may reduce willingness to continue supplementation. These factors may limit the feasibility of incorporating garlic preparations into prolonged therapeutic regimens.

### 9.8 Regulatory Considerations

Regulatory differences further complicate the clinical use of garlic‐based products. In many countries, garlic preparations are marketed as dietary supplements or nutraceuticals rather than pharmaceutical products, resulting in differences in requirements for manufacturing quality, labeling, quality control, and demonstration of clinical efficacy. Consequently, commercial products may differ in purity, potency, and consistency. Appropriate regulatory oversight and adherence to good manufacturing practices are therefore important for ensuring the quality and reliability of garlic‐based products intended for clinical use.

## 10 Limitations and Future Directions

The clinical evidence for garlic remains constrained by differences in study design, sample size, participant characteristics, follow‐up duration, and outcome assessment. The evidence base is also uneven across clinical indications, with some therapeutic applications supported by considerably more evidence than others. Variation in endpoint definitions and analytical approaches further limits direct comparison among investigations. These methodological limitations reduce the certainty of clinical conclusions and restrict their generalizability across populations and disease settings. Future clinical research should prioritize adequately powered, multicenter randomized controlled trials addressing clearly defined therapeutic questions. Studies should incorporate rigorous allocation procedures, appropriate eligibility criteria, validated patient‐relevant outcomes, prespecified analytical methods, and comprehensive adverse‐event assessment. Longer observation periods should be incorporated when necessary to determine whether observed effects are sustained and clinically meaningful.

Another priority is to clarify heterogeneity in individual treatment responses. Future investigations should examine whether demographic and clinical characteristics, genetic factors, concomitant therapies, or gut microbiome profiles contribute to differences in therapeutic response. Integration of pharmacogenomic and microbiome approaches with clinical research may help identify patient populations with distinct patterns of benefit or adverse effects. The methodological quality of future evidence syntheses should also be strengthened. Reviews should distinguish findings according to study design and clinical indication, critically assess methodological quality and risk of bias, and systematically investigate sources of statistical and clinical heterogeneity. Conflicting findings should be examined in relation to differences in populations, interventions, outcomes, and study methods rather than simply summarized. Quantitative synthesis should be performed only when the available studies are sufficiently comparable to support valid interpretation.

Finally, experimental findings should be interpreted according to their evidentiary level. Molecular and preclinical studies can establish biological plausibility and identify potential therapeutic mechanisms, but they cannot independently demonstrate clinical effectiveness. Future investigations should therefore directly evaluate whether experimentally identified mechanisms are associated with clinically meaningful patient outcomes. This evidence‐based approach will help establish which therapeutic applications of garlic are supported by sufficient human evidence and distinguish promising biological effects from clinically demonstrated benefits.

## 11 Limitations of This Review

Most included studies were short‐term and had small sample sizes, limiting statistical power and reducing the ability to draw robust, generalizable conclusions. The short follow‐up periods also prevented assessment of long‐term therapeutic effects and safety of garlic supplementation. There was substantial heterogeneity across studies in design, garlic formulations (raw garlic, aged garlic extract, garlic oil, and standardized supplements), dosing regimens, and intervention duration. Outcome measures also varied widely, particularly for cardiometabolic, antimicrobial, and anti‐inflammatory effects. This heterogeneity limited comparability and prevented quantitative synthesis or meta‐analysis.

A considerable proportion of evidence came from preclinical studies (in vitro and animal models). Although these provide important mechanistic insights, their translational relevance is limited due to species differences in metabolism, absorption, and pharmacodynamics, making direct clinical extrapolation uncertain. Several studies also had methodological weaknesses, including inadequate randomization, lack of blinding, and absence of placebo controls. These issues increase the risk of selection, performance, and detection bias, potentially leading to overestimation of treatment effects. Finally, important clinical aspects were inconsistently reported or absent, including long‐term safety, bioavailability of active sulfur compounds, and potential herb–drug interactions. This represents a key evidence gap, particularly for patients using garlic alongside conventional pharmacotherapy

## 12 Conclusion

*Allium sativum* L. is a phytochemically rich medicinal plant with broad pharmacological activities supported by clinical and preclinical evidence. Its effects are mainly linked to organosulfur compounds derived from alliin, including allicin, diallyl sulfides, ajoene, vinyldithiins, and stable aged‐garlic compounds such as S‐allyl cysteine and S‐allyl mercaptocysteine. Other constituents, including phenolics, flavonoids, saponins, fructans, vitamins, and trace elements, contribute synergistically to its biological effects. Clinical evidence supports benefits in cardiovascular and metabolic outcomes, including blood pressure, lipid profiles, glycemic control, oxidative stress, and inflammation. Additional potential effects have been reported in metabolic syndrome, PCOS, NAFLD, osteoarthritis, and endometriosis. Preclinical studies further demonstrate renoprotective, hepatoprotective, immunomodulatory, antimicrobial, antiparasitic, anticancer, and antimalarial activities through antioxidant defense, cytokine modulation, nitric oxide regulation, Nrf2 signaling, apoptosis, and tissue repair pathways. However, evidence interpretation remains limited by variability in garlic formulations, bioactive compound standardization, dosage, duration, populations, and study quality. Although findings are promising, inconsistent clinical outcomes and the reliance on preclinical evidence indicate that garlic should be considered a promising nutraceutical and complementary agent rather than an established therapy. Future studies should focus on standardized preparations, phytochemical validation, long‐term safety, and large, well‐designed clinical trials to define its therapeutic role.

## Author Contributions

**Gudisa Bereda**: conceptualization, visualization, methodology, data curation, investigation, validation, project administration, supervision, resources, writing – original draft, writing – review & editing. The author checked and confirmed the final version of the manuscript.

## Funding

The author has nothing to report.

## Ethics Statement

This article does not contain any studies involving human participants or animals performed by the author.

## Conflicts of Interest

The author declares no conflicts of interest.

## Supporting information

Supplementary Material

## Data Availability

No data were used for the research described in the article.
