# Supplementary material for: Pharmacological and Therapeutic Potential of Garlic (Allium sativum): Integrating Phytochemistry, Molecular Mechanisms, and Clinical and Preclinical Evidence
Source: ChemistryOpen. 2026 Oct 27;15(11):e70321. doi: 10.1002/open.70321 (PMC13714450; doi:10.1002/open.70321)
Supplement: Supplementary file 1 [file OPEN-15-e70321-s001.pdf]

Supplementary Figure 1: SANRA—a scale for the quality assessment of narrative review articles

1) Justification of the article's importance for the readership

The importance is not justified ____0

The importance is alluded to, but not explicitly justified_________________________1

The importance is explicitly justified____2

Score: 2

2) Statement of concrete aims or formulations of questions

No aims or questions are formulated_______________________________________0

Aims are formulated generally but not concretely or in items of clear questions _________1

One or more concrete aims or questions are formulated ________________________2

Score: 2

3) Description of the literature search

The search strategy is not presented _______________________________________0

The literature search is described briefly ____________________________________1

The literature search is described in detail, including search terms and inclusion criteria _______2

Score: 2

4) Referencing

Key statements are not supported by references ____________________________0

The referencing of key statements is inconsistent ____________________________1

Key statements are supported by references ________________________________2

Score: 2

5) Scientific reasoning (e.g., incorporation of appropriate evidence, such as RCTs in clinical medicine)

The article's point s not based on appropriate arguments ________________________0

Appropriate evidence is introduced selectively _________________________________1

Appropriate evidence is generally present ____________________________________ 2

Score: 2

6) Appropriate presentation of data (e.g., absolute vs relative risk; effect sizes without confidence intervals)

Data are presented inadequately ____0

Data are often not presented in the most appropriate way___________________________1

Relevant outcome data are generally presented appropriately ______________________

Score: 2

Summary: 12/12
